# Higher prevalence of geriatric depression, catastrophizing pain and sleep disorders in institutionalized elders: a cross-sectional study in Galle District, Sri Lanka

**DOI:** 10.1186/s12877-021-02536-9

**Published:** 2021-12-07

**Authors:** N. W. B. Y. Abeysekera, Eric De Zoysa

**Affiliations:** 1 Department of Nursing, Faculty of Allied Health Sciences, University of Ruhuna, Mahamodara, Galle, Sri Lanka; 2 Department of Biochemistry, Faculty of Medicine, University of Ruhuna, Karapitiya, Galle, Sri Lanka

**Keywords:** Geriatric depression, Catastrophizing pain, Sleep disorders, Sleep quality, Correlation, Institutionalized elders

## Abstract

**Background:**

Population aging is a significant social problem in the twenty first century. Recent economic and social changes lead increasing number of elders to spend their lives in elderly homes. Institutionalized elders have to face many physical and psychological problems which negatively impact their quality of life. Geriatric depression (GD), catastrophizing pain (CP) and sleep disorders (SD) are some common problems among them.

**Methods:**

Present study was designed to assess the prevalence of GD, CP and SD and their correlations in institutionalized elders. A descriptive cross-sectional study was conducted in elderly homes (*n* = 20) in the Galle district of Sri Lanka enrolling 310 subjects. GD, CP and SD were assessed using validated Sinhala versions of Geriatric Depression Scale (GDS), Pain Catastrophizing Scale (PCS) and Pittsburgh Sleep Quality Index (PSQI) respectively. Data were analyzed using SPSS version 25.0 for windows by using descriptive statistics, the Pearson’s chi-square test and Pearson’s bivariate correlation (*p* < 0.05).

**Results:**

Among the participants (response rate: 95.7%), 34.8% (*n* = 108) and 65.2% (*n* = 202) were males and females respectively. Age range of the subjects was 60–103 years with the mean age of 74.97 years (SD 8.852). Most of the study subjects (*n* = 234, 75.5%) had spent five or less than 5 years in elderly homes at the time of the study and 52.8% (*n* = 164) of them were unmarried. GD was present in 76.5% (95% CI: 71.7–81.2) of subjects and of them 44% had moderate to severe depression. PCS revealed that 29% (95% CI: 24.0–34.1) had CP. SD were identified in 55.5% (95% CI: 49.5–61.0) of elders and according to PSQI, 86% (95% CI: 82.3–90.0) had poor quality sleep. Positive correlations between GD and CP (*r* = 0.24, *p* < 0.01), GD and SD (*r* = 0.13, *p* = 0.02), CP and SD (*r* = 0.32, *p* < 0.01) were statistically significant.

**Conclusions:**

Prevalence of GD, CP and SD were significantly higher in this sample of institutionalized elders who were apparently healthy. Findings highlighted the importance of early screening of physical and psychological problems in institutionalized elders to assure better quality of life and to reduce the burden to health care system of the country.

## Introduction

Population ageing is considered as one of the most significant social transformations of the twenty-first century [1]. The population of the world’s older adults is estimated to be almost double from 12 to 22% between 2015 and 2050 [2]. As a result of the increase elderly population, so many health, financial and social challenges are emerging all over the world. Aging was not a significant issue in Sri Lankan society a few decades back because life expectancy was not high and they had an extended family structure where they had large number of family members to take care of the aged [3]. With rapid economic and social changes, the family structure of Sri Lanka has gradually transformed to a nuclear family where the capacity of the family to look after their elderly has been declined [4] and this has led many of them to spend their remaining lives as institutionalized elders.

Mental health and physical well- being are as important in older age as at any other time of life. However, according to WHO more than 20% of adults aged 60 years and over suffer from a mental and/or a neurological disorder [2]. It is a well-known fact that institutionalized elders face so many financial, physical, emotional and psychological problems other than to the burden of living away from their family. Geriatric depression is one of the commonest mental problems in elderly population and institutionalization was considered as one of the most important risk factors for depression among older people. If it is not detected and treated, common subsyndromal depression among elders can lead to major depression and critical consequences. Prevalence of depression among males and females are above 5.5 and 7.5% respectively in 55–74 year- age group and peak level can be seen in older adulthood [5]. As WHO implies, symptoms of depression are often overlooked and untreated because they co-exist with other problems encountered by older adults. Further, it was identified that individuals living in nursing homes, in the United States had a higher prevalence of depression (15–25%) when compared to the prevalence of depression among older individuals seen at primary healthcare clinics (5%) [6]. A study conducted among community dwelling adults aged ≥60 years in one divisional secretariat of Kandy District revealed that the prevalence of depression was 31.8% [7] and a study conducted in Sri Lanka in 2009 found that overall prevalence of depressive symptoms among community dwelling adults over 60 years was 27.8% [8]. Findings of these research indicated that the prevalence of depression among elderly population in Sri Lanka is considerably high. Even though the depression is a significant health problem at present, there is a huge potential for it to become a neglected public health burden among elderly [9].

Pain is an unpleasant sense of discomfort which is also a common complaint that comes with old age and it may persist or progress over a long period of time. Unlike acute pain which arises suddenly in response to a specific injury which is usually treatable, chronic pain persists over time and is often resistant to medical treatments [10]. Pain catastrophizing is an exaggerated negative response to actual or anticipated pain characterized by worry, fear, and difficulty directing attention away from pain and this is one of the most reliable predictors of the chronic painful experience [11–13].

Sleep disorders are a group of conditions that disturbs the regular sleep pattern. Insomnia, sleep apnea, parasomnias, restless leg syndrome and narcolepsy are the most common sleep disorders and depending on the type of disorder people may experience negative impacts on energy, mood, concentration and overall health [14]. According to the estimations, sleep disturbances affect more than 50% of community dwelling individuals and two-thirds of institutionalized elders aged 65 years or above [15]. Sleep disorders and catastrophizing pain are some of the important problems which are highly prevalent among institutionalized elders and neglected most of the time [16]. These issues need more attention of caregivers to maintain the quality of life in institutionalized elders.

Compared to the other South Asian countries, Sri Lanka has a higher proportion of older population. Ageing population (over 60 years of age) in Sri Lanka is2.5 million and that is about 12.5% of the total population of the country and according to the current trend this percentage may increase further [17]. From the twenty-five districts in Sri Lanka, Colombo, Galle, Matara and Kegalle districts have been identified as areas with a high ageing index [18]. On this background, it is important to identify the present status of psychological and other psychosomatic problems of institutionalized elderly population in Sri Lanka to plan the future interventions to make their quality of life better. Therefore, the present study was designed to assess the prevalence of depression, catastrophizing pain, sleep disorders and their correlations among institutionalized elders who are apparently healthy in the Galle district, Sri Lanka.

## Material and methods

A descriptive cross-sectional study was conducted in all (*n* = 20) elderly homes in the Galle district of Sri Lanka which were registered under the Social Services Department from August 2019 to November 2019. Ethical approval for the study was obtained from the Ethical Review Committee of the Faculty of Allied Health Sciences, University of Ruhuna, Galle, Sri Lanka. All the institutions for the elderly people in the Galle District were selected for the study. All the elders 60 years and older and who were living in elderly homes for more than a year at the time of the study, were recruited by using a convenience sampling method. Elders who were living in an elderly home for less than 1 year, aged less than 60 years, those who were diagnosed with neurological illnesses, major psychiatric illnesses, depression and malignancy were excluded from the study after confirming with the diagnosis card or inquiring about the past medical history and long-term drug treatment that they were on. Subjects who are bedridden and severely ill and subjects with severe visual or hearing impairment were also excluded from the study after individual assessment by the investigator. Informed written consent was obtained from each of the participants who were willing to participate in the study.

In this study all the data were obtained through face-to-face interviews by the investigator using a pretested interviewer administered questionnaire. There were 05 Tamil nationals in the study sample and they were fluent in Sinhala since Galle district is mostly a Sinhala language speaking area. Data on basic information including socio demographic data, education, substance usage, satisfaction about the elderly home, period of stay and details on health condition were collected.

When data was collected, there were 23 elderly homes in Galle District registered under the Social Service Department [19]. One elderly home was closed by the time of data collection, one elderly home was reserved for Buddhist clergy and one for mentally handicap elders. Permission was not granted to collect data from the elderly home reserved for Buddhist clergy and data collection from the home for mentally handicap elders was omitted. Therefore, all the remaining 20 elderly homes were included in the study. Based on exclusion criteria,78 subjects were excluded from the study. Therefore 324 eligible subjects were identified and of those 310 subjects consented to participate in the study.

Data on depression was collected using the validated Sinhala form of the short version of Geriatric Depression Scale (GDS) [20, 21]. The short version of GDS consists of 15 items and it indicated the presence of depression when answered positively while the rest of the questions (numbers 1, 5, 7, 11, 13) indicated depression when answered negatively. Depending on age, education and complaints, scores of 0–4 are considered normal, 5–8 indicate mild depression, 9–11 indicate moderate depression and 12–15 indicate severe depression. The validated Sinhala version of GDS was found to have 73.3% of sensitivity and specificity when evaluated against diagnostic criteria [21].

Validated Sinhala version of Pain Catastrophizing Scale (PCS) [22, 23] was used to collect data about physical and psychological pain. PCS is used to quantify the catastrophic thinking related to pain. The PCS instructions ask participants to reflect on past painful experiences, and to indicate the degree to which they experienced each of 13 thoughts or feelings when experiencing pain, on 5-point scales with the end points 0 = not at all and 4 = all the time. The PCS yields a total score and three subscale scores assessing rumination, magnification and helplessness. The PCS total score is computed by summing responses to all 13 items. PCS total scores range from 0 to 52 and > 20 of score indicates a considerable catastrophizing pain. The PCS subscales are computed by summing the responses to the following items: Rumination: Sum of items 8, 9, 10 and 11, Magnification: Sum of items 6, 7 and 13, Helplessness: Sum of items 1, 2, 3, 4, 5, and 12.

Pittsburgh Sleep Quality Index (PSQI, validated Sinhala version) [24] was used to collect data on sleep of the elders and for both validated Sinhala versions were used. PSQI is a widely used standardized instrument to assess sleep quality in clinical and research settings. It was developed as a standardized measure of sleep quality which can be used in clinical practice. The PSQI assesses sleep quality during the previous month. It consists of 19 self-rated questions which are scored to obtain a total score. The 19 items are grouped into seven components which are added to give the total score. The range of score is 0–21. Higher scores indicate worse sleep quality. The validated Sinhala version of PSQI has good internal consistency (Cronbach’s alpha = 0.85).

In data analysis, descriptive data were presented as mean and standard deviation (SD) or median unless stated otherwise. Frequencies and crosstabs were used to assess the prevalence of depression, catastrophizing pain and sleep disorders as numerical and categorical variables. Pearson’s bivariate correlation model was used when examining the association between depression and other variables recorded as continuous numerical variables. Pearson’s Chi square test was used to detect differences in categorical variables. Two-tailed *p* value less than 0.05 was considered as the level of statistical significance (*p* < 0.05).

## Results

### Socio-demographic characteristics of study population

In total, 310 institutionalized elders were enrolled in the study with a response rate of 95.7% and the age range of the subjects was 60–103 years with the mean (SD) age of 74.97 (8.85) years. There were 108 (34.8%) males and 202 (65.2%) females in the sample. Majority of the subjects (52.8%) were unmarried and 32.3 and 11.3% of them were married and widowed respectively. Only 1% of the elders got higher education and 36.8 and 37.4% of them had primary and secondary education respectively. Seventy percent of the subjects were previously employed and the period of stay in elderly home in the majority of the subjects (75.5%) was one to five years. When it was inquired about their perception about the services and facilities of elderly homes, 82% of them were satisfied about the services provided.

Usage of substance by the study subjects was assessed in the study and beetle (45.2%) was the commonly used substance other than cigars (21.6%) and alcohol (26.1%). Majority of the study subjects used any kind of substances 180 (58.1%) at least once in their lifetime and 106 (34.2%) subjects used one substance and 74 (23.9%) used more than one substance. When considering the health status of the study subjects, most of them (92.6%) had at least one comorbidity at the time of the study and vision impairment (*n* = 256,82.6%) and hypertension (*n* = 99, 31.9%) were the most prevalent comorbidities (Table 1).

**Table 1:** Frequency distributions of socio-demographic characteristics of the institutionalized elders in Galle District

	Frequency (n)	Percentage (%)
Age subgroup		
60-74Y Young Old	155	50.0
75-84Y Middle Old	110	35.5
> 85Y Old Old	45	14.5
Gender		
Female	202	65.2
Male	108	34.8
Religion		
Buddhist	292	94.2
Christian	13	4.2
Hindu	5	1.6
Ethnicity		
Sinhala	305	98.4
Tamil	5	1.6
Marital status		
Single	164	52.8
Married	100	32.3
Divorced	7	2.3
Separated	4	1.3
Widowed	35	11.3
Educational level		
No schooling	41	13.2
Primary education	114	36.8
Secondary education	116	37.4
Up to O/L	31	10.0
Up to A/L	5	1.6
Higher education	3	1.0
Previous employment		
Not employed	92	29.7
Employed	218	70.3
Years of boarding		
1-5 years	234	75.5
6–10 years	49	15.8
> 10 years	27	8.7
Satisfaction of the elderly home		
Not satisfied	55	17.7
Satisfied	255	82.3

### Prevalence of depression and associated socio-demographic factors

**Fig. 1:**
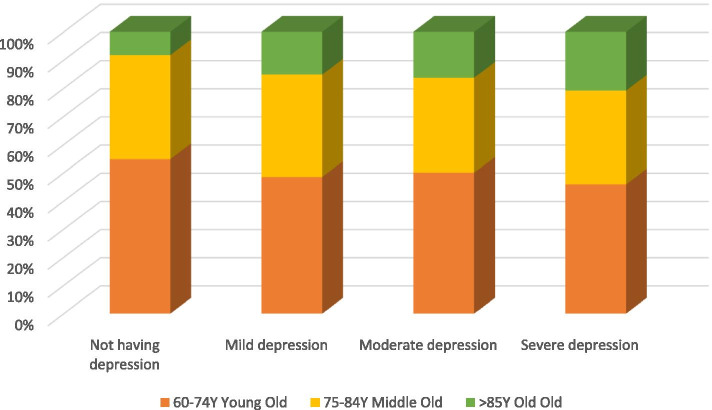
Severity of depression among age sub groups of institutionalized elders in the Galle District

Geriatric depression was present in 237 (76.5, 95% CI: 71.7–81.2) subjects and from them 153(64.6%) were females and 84 (35.4%) were males. Figure 1 illustrate the severity of depression among the age subgroups of the study subjects. From the study subjects who had depression, 132 (55.7%) had mild depression, 80 (33.8%) and 25 (10.5%) had moderate and severe depression respectively. Further, it was found that prevalence of geriatric depression was very high (76.2%) among elders who were unmarried.

### Prevalence of catastrophizing pain in elderly

From the study subjects, when it was asked about their experience of pain during last 6 months 244 (78.7%) responded that they had experienced some kind of a pain and from them 161 (66%) were females and 83 (34%) were males. Thirty eight percent of them (*n* = 93) had chronic pain (i.e., pain lasting more than 3 months). According to the scores of the pain catastrophizing scale, 285 (91.9%) of study subjects had catastrophizing pain (study subjects who got scores1–52) and among them 195 (68%) did not have a considerable catastrophizing pain (scores 1–20). However, in this study sample 90 (29, 95% CI: 24.0–34.1) had catastrophizing pain. Majority of the subjects (42.2%) who had moderate to severe catastrophizing pain belonged to 60–74-year age subgroup and 30 and 27.8% of them belonged to age subgroups 75–84 years and > 85 years respectively. Fifty-five (61.1%) females and 35 (38.9%) males had moderate to severe catastrophizing pain and 29% of them had chronic catastrophizing pain. According to the subscales of the PCS, it was found that from the total study subjects, 241 (77.7%) had helplessness, 257 (82.9%) had magnification and 260 (83.9%) had rumination.

### Prevalence of sleep disorders and assessment of quality of sleep

According to the response of the study subjects to the general question of whether they had a sleep problem or not, 172 (55.5, 95% CI: 49.5–61.0) subjects responded that they had experienced problems with sleep and among them 107 (62.2%) were females and 65 (37.8%) were males.

However, analysis of the quality of sleep using PSQI showed that only 43 (13.9%) had overall good quality sleep and 267 (86.1, 95% CI: 82.3–90.0)) had poor quality sleep. From those who had poor quality sleep 64.4% were females and 35.6% were males.

Most of the study subjects 173 (55.8%) had fairly good subjective sleep quality, 268 (86.5%) had sleep latency and only 93 (30%) had more than 7 h of sleep per day. Majority of subjects (*n* = 299, (96.5%) had sleep disturbances less than once a week and 292 (94.2%) had not used sleep medications for the last 1 month prior to the data collection, and 240 (77.4%) did not have any kind of daytime dysfunction according to the scoring of subscale indicators of PSQI (Table 2).

**Table 2:** Frequency distribution of results of PSQI sub scales among institutionalized elders in the Galle District

PSQI sub scale	Frequency (n)	Percentage (%)
Subjective sleep quality		
Very good	57	18.4
Fairly good	173	55.8
Fairly bad	56	18.1
Very bad	24	7.7
Sleep latency		
No sleep latency	42	13.5
Having sleep latency	268	86.5
Sleep duration		
> 7 h	93	30.0
6–7 h	63	20.3
5–6 h	67	21.6
< 5 h	87	28.1
Habitual sleep efficiency		
> 85%	99	31.9
75–84%	72	23.2
65–74%	40	12.9
< 65%	99	31.9
Sleep disturbance		
Not during the past month	11	3.5
Less than once a week	299	96.5
Use of sleeping medication		
Not during the past month	292	94.2
Less than once a week	4	1.3
Once or twice a week	9	2.9
Three or more times a week	5	1.6
Daytime dysfunction		
No daytime dysfunction	70	22.6
Has daytime dysfunction	240	77.4

### Correlations

According to the results of Pearson’s’ bivariate correlation there was a statistically significant positive correlation between geriatric depression and presence of catastrophizing pain (*r* = 0.24, *p* < 0.01) and its severity (*r* = 0.23, *p* < 0.01). The correlation between severity of depression & sleep disorders was also significant (*r* = 0.13, *p* = 0.02) and further, there was a significant positive correlation between catastrophizing pain and sleep disorders (*r* = 0.32, *p* < 0.01). However, the positive correlation between geriatric depression and sleep disorders was not significant. Furthermore, there was a positive statistically significant correlation between overall quality of sleep (according to the global score of the PSQI) and chronic catastrophizing pain (*r* = 0.13, *p* = 0.02).

## Discussion

Population aging is a common phenomenon across advanced economies but it is more significant in Sri Lanka, being one of the fastest aging countries in South Asia. A rapid rise expected in the share of population over 65 years in Sri Lanka’s total population from 9.4% in 2015 to 21% by 2045 and to 35.6% by 2100 and these changes in population age structure will result in a rapid rise in the dependency ratio as well [25]. With the gradual increase in elderly population, society will have to face many problems in looking after them while maintaining their quality of life. Even though the authorities take various steps to maintain the physical wellbeing of the elders by means of providing food, accommodation and other essentials, psychological wellbeing is mostly neglected in elders especially those who are in elderly homes. Elderly population growth and the increasing number of institutionalized elders has become a rising trend in Sri Lanka as well and the present study assessed some of the important physical and psychological problems in institutionalized elders in one of the districts which has a high ageing index.

According to socio-demographic data, majority of the study sample were females. According to the estimated mid-year population in 2019 by the Department of Census and Statistics Sri Lanka, female population of age group of 60–64 years was 53.6% and in age group over 75 years it was as high as 59.6% [26] Older adults prefer elderly homes as they give them security and medical attention. They may experience loneliness but at the same time feel a sense of independence [27]. In this sample also most of the study subjects were satisfied with the facilities and services provided by the institutions and when it was inquired, the reason for their positive answer was that most of them did not have any other option rather than staying in elderly homes.

A study done in Sri Lanka revealed that the prevalence of depression among the institutionalized elders in Colombo district was 56% and of them 23.2% had severe depression [28]. The overall prevalence of depression is higher (76.5%) in the present study when compared to that study but the percentage of severe depression is less (10.5%). Comparatively same findings were identified in a study which was conducted in India and it revealed that, about one third of the elderly population of India suffered from depression with female preponderance [29]. Though Asian countries with similar backgrounds had similar results and the findings from other non-Asian countries were slightly different. A Brazilian study revealed that 49.76% of the elderly in Brazil and in 61.40% of the Portuguese seniors had depressive symptoms [30] and it is obvious that a significant proportion of elders had depression in many other countries in the world as well [31–34].

Analysis of catastrophizing pain reveled that even though a vast majority of subjects had catastrophizing pain, only 29% of study subjects were suffering from moderate to severe chronic catastrophizing pain and most of the subjects in this study sample had helplessness, magnification and rumination according to the subscales of the PCS. A study conducted in Sweden to assess catastrophizing pain in elderly had similar results to the present study and the prevalence of catastrophizing pain in that sample was 30.9% and the same study identified catastrophizing pain as a potent predictor of negative pain-related outcomes in general [35].

Presence of poor-quality sleep, in majority of the subjects (86.1%) in this study is supported by the findings of other studies done on elderly in nursing homes in China (67.3%) and on elderly in Sao Paulo (63.2%) [36, 37]. However, prevalence of poor-quality sleep is relatively higher in this sample of elders. Furthermore, some previous researches which was conducted in recent past revealed that accelerated age-related changes in sleep architecture may be linked to depressed mood in older adults and symptoms of depression, fatigue, and insomnia were more severe in subjects with moderate-to extreme pain interference than in those who reported less pain [38, 39].

Results of the present study found out important correlations between the study parameters and according to that there was a significant positive correlation between geriatric depression, its severity and the catastrophizing pain, a significant positive correlation between overall quality of sleep and chronic catastrophizing pain, a positive correlation between severity of depression and sleep disorders and also a significant positive correlation between catastrophizing pain and sleep disorders. These findings highlighted that the physical and psychological problems of the institutionalized elders are inter- related and overall approach is mandatory in addressing these issues. In comparison, a study which was conducted in Sri Lanka in 2016 found that there was a positive correlation between geriatric depression and chronic pain but it was not statistically significant. However, results of that study indicated that, pain and related presenting complaints were significantly associated with depression [24]. When comparing the findings with the Asian studies there are quite similar findings to the present study and according to a study which was conducted in Hong Kong, pain catastrophizing was found to partially mediate the connection between pain intensity and depressive symptoms [40].

## Conclusions

Prevalence of depression, sleep disorders and presence of catastrophizing pain were high in this sample of institutionalized elders who were considered as “healthy” before. Early detection of those problems enables to prevent them from going into more severe consequences and early intervention will help to improve the quality of life of them and to reduce the long-term health burden to the society and the country. Positive correlations between the physical and psychological issues highlights the importance of a holistic approach of the management of problems in institutionalized elders.

### Limitations of the study

In this study paid elderly homes were not included and socio-economic status of inmates in these two types of elderly homes are different and it interfere with the representativeness of the sample. Further, 19.4% (*n* = 78) subjects were excluded according to the exclusion criteria in this study and there is a potential underestimation of the prevalence values of the main outcome measures due to this exclusion.

## Data Availability

The datasets generated and/or analyzed during the current study are not publicly available due to the agreement of confidentiality but are available from the corresponding author on reasonable request.

## Abbreviations

GD: Geriatric Depression
CP: Catastrophizing Pain
SD: Sleep Disorders
GDS: Geriatric Depression Scale
PCS: Pain Catastrophizing Scale
PSQI: Pittsburgh Sleep Quality Index

## References

[1] United Nations (2019). World population ageing 2019: highlights. Department of Economic and Social Affairs, Population Division.

[2] WHO. Mental health of older adults. World Health Organization. 2017. http://www.who.int/news-room/fact-sheets/detail/mental-health-of-older-adults. Accessed 13 Feb 2020.

[3] De Silva N (2006). Female migration: gender and restructuration.

[4] Weeratunga KM, Hugo G. Changing Family Structure in Sri Lanka. Sri Lanka J Popul Stud. 2014.

[5] WHO (2017). Global strategy and action plan on ageing and health.

[6] Arnold JF, James B, Alan SB, et al. NIH consensus development panel on depression in late life diagnosis and treatment of depression. JAMA. 1992. 10.1001/jama.1992.03490080092032.

[7] Khaltar A, Priyadarshani N, Delpitiya N, Jayasinghe C, Jayasinghe A, Arai A, et al. Depression among older people in Sri Lanka: with special reference to ethnicity: depression among older Sri Lankans. Geriatr Gerontol Int. 2017;17. 10.1111/ggi.13090. 10.1111/ggi.13090 28776918

[8] Malhotra R, Chan A (2010). Ostbye. T. Prevalence and correlates of clinically significant depressive symptoms among elderly people in Sri Lanka: findings from a national survey. Int Psychogeriatr.

[9] Buvneshkumar M, John KR, Logaraj MA (2018). Study on prevalence of depression and associated risk factors among elderly in a rural block of Tamil Nadu. Indian J Public Health.

[10] Shiel Jr., W. C. Medical definition of chronic pain. MediciNet. 2018. https://www.medicinenet.com/script/main/art.asp?articlekey=22430. Accessed 15 Feb 2020.

[11] Wells NM, Rollings KA, Ong AD, Reid MC. Nearby nature buffers the pain Catastrophizing–pain intensity relation among urban residents with chronic pain. Front Built Environ. 2019:5–142. 10.3389/fbuil.2019.00142 PMC10358861 37475721

[12] Turk DC, Rudy TE (1992). Cognitive factors and persistent pain: a glimpse into Pandora's box. Cogn Ther Res.

[13] Sullivan MJ, Thorn B, Haythornthwaite JA, Keefe F, Martin M, Bradley LA, et al. Theoretical perspectives on the relation between catastrophizing and pain. Clin J Pain. 2001;17:1.52–1.64. 10.1097/00002508-200103000-00008. 10.1097/00002508-200103000-00008 11289089

[14] Roddick J, Cherney K (2016). What Are Sleep Disorders? Sleep Disorders.

[15] Harrington JJ, Chiong TL (2007). Sleep and older patients. Clin Chest Med.

[16] de Silva RM, Afonso P, Fonseca M, Teodoro T (2020). Comparing sleep quality in institutionalized and non-institutionalized elderly individuals. Aging Ment Health.

[17] LBO. Sri Lanka’s ageing population poses socio-economic challenges. Watch Tower. 2016. www.lankabusinessonline.com. Accessed 16 Feb 2020.

[18] Maduwage S. Sri Lankan “silver age” population. J College Commun Phys Sri Lanka. 2019:1–2.

[19] Southern Provincial Department of Social Welfare, Probation & Childcare Services. https://www.socialservicedept.sp.gov.lk/index.php?option=com_content&view=article&id=13&Itemid=128&lang=en#galle-district-elders-home. Accessed 18 Oct 2019.

[20] Yesavage JA, Brink TL, Rose TL, Lum O, Huang V, Adey M (1983). Development and validation of a geriatric depression screening scale: a preliminary report. J Psychiatr Res.

[21] Kulathunga M, Umayal S, Somaratne S, Srikanth S, Kathriarachchi S, De Silva KRD (2010). Validation of the geriatric depression scale for an elderly Sri Lankan clinic population. Indian J Psychiatry.

[22] Sullivan MJL (2009). PCS the pain catastrophizing scale user manual.

[23] Pallegama RW, Ariyawardana A, Ranasinghe AW, Sitheeque M, Glaros AG, Dissanayake WP, et al. The Sinhala version of the pain catastrophizing scale: validation and establishment of the factor structure in pain patient and healthy adults. Pain Med. 2014;15. 10.1111/pme.12529. 10.1111/pme.12529 25105529

[24] Anandakumar D, Ranatunga SS, Dayabandara M, Hanwella R, De Silva VA (2016). Validation of the Sinhala version of the Pittsburgh sleep quality index. Ceylon Med J.

[25] Asian Development Bank. Growing Old Before Becoming Rich Challenges of An Aging Population in Sri Lanka. 10.22617/TCS190612-2. Accessed 5 Jan 2020.

[26] Department of Census and Statistics Sri Lanka. http://www.statistics.gov.lk/GenderStatistics/StaticalInformation/Population. Accessed 5 Jan 2020.

[27] Menezes S, Thomas TM (2018). Status of the elderly and emergence of old age homes in India. Int J Soc Sci Manage.

[28] Wijeratne MDM, Wijerathne SA, Wijesekara SG, Wijesingha I. Prevalence of depression among institutionalized elders in the Colombo district. Stud Med J Facult Med Colombo. 2010:27–30.

[29] Pilania M, Yadav V, Bairwa M (2019). Prevalence of depression among the elderly (60 years and above) population in India, 1997–2016: a systematic review and meta-analysis. BMC Public Health.

[30] Leal MCC, Apóstolo JLA, Mendes AMDOC, Marques APDO (2014). Prevalence of depressive symptoms and associated factors among institutionalized elderly. Acta Paulista Enfermagem.

[31] Malhotra R, Chan A, Østbye T (2010). Prevalence and correlates of clinically significant depressive symptoms among elderly people in Sri Lanka: findings from a national survey. Depressive symptoms among Sri Lankan elderly international Psychogeriatrics. Int Psychogeriatr Assoc.

[32] Sarin K, Punyaapriya P, Sethi S, Nagar I (2016). Depression and hopelessness in institutionalized elderly: a societal concern. Open J Depression.

[33] Silva ER, Sousa ARP, Ferreira LB, Peixoto HM (2012). Prevalence and factors associated with depression among institutionalized elderly individuals: nursing care support USP. Rev Esc Enferm.

[34] Santiago LM, Mattos IE (2014). Depressive symptoms in institutionalized older adults. Rev Saúde Pública.

[35] Dong HJ, Gerdle B, Bernfort L, Levin LÅ, Dragioti E (2020). Pain catastrophizing in older adults with chronic pain: the mediator effect of mood using a path analysis approach. J Clin Med.

[36] Zhu X, Hu Z, Nie Y (2020). The prevalence of poor sleep quality and associated risk factors among Chinese elderly adults in nursing homes: a cross-sectional study. PLoS One.

[37] Araújo CLO, Ceolim MF (2010). Sleep quality of elders living in long-term care institutions. Rev Esc Enferm USP.

[38] Palagini L, Baglioni C, Ciapparelli A, Gemignani A, Riemann D. REM sleep dysregulation in depression: state of the art. Sleep Med Rev. 2013:17.377–90. 10.1016/j.smrv.2012.11.001 23391633

[39] Smagula SF, Reynolds CF, Ancoli-Israel S (2015). Sleep architecture and mental health among community-dwelling older men. J Gerontol B Psych Sci Soc Sci.

[40] Cheng ST, Leung CMC, Chan KL, Chen PP, Chow YF, Chung JWY, et al. The relationship of self-efficacy to catastrophizing and depressive symptoms in community-dwelling older adults with chronic pain: a moderated mediation model. PLoS One. 2018;13:9. 10.1371/journal.pone.0203964. Accessed 16 Feb 2020. 10.1371/journal.pone.0203964 PMC6143242 30226892

